# Exploring the dynamics of Programmed Death-Ligand 1 in canine lymphoma: unraveling mRNA amount, surface membrane expression and plasmatic levels

**DOI:** 10.3389/fvets.2024.1412227

**Published:** 2024-07-26

**Authors:** Alessandra Ubiali, Luiza Cesar Conti, Paola Dall’Ara, Raffaella De Maria, Luca Aresu, Pierangelo Moretti, Federica Sini, Fulvio Riondato, Damiano Stefanello, Stefano Comazzi, Valeria Martini

**Affiliations:** ^1^Department of Veterinary Medicine and Animal Sciences, University of Milan, Lodi, Italy; ^2^Department of Veterinary Sciences, University of Turin, Grugliasco, TO, Italy

**Keywords:** Programmed Death-Ligand 1, lymphoma, dog, flow cytometry, polymerase chain reaction, immunoassay

## Abstract

**Introduction:**

Programmed Death-Ligand 1 is a well-known immune checkpoint molecule. Recent studies evaluated its expression in different canine cancer types through different laboratory techniques. The present study aims to evaluate the surface membrane protein expression (mPD-L1) by means of flow cytometry (FC) in different canine lymphoma immunophenotypes. Furthermore, in a subset of cases, mRNA and plasmatic soluble protein (sPD-L1) have been assessed in the same patient, and correlations among results from the three analyses investigated.

**Methods:**

Samples obtained for diagnostic purpose from untreated dogs with a confirmed lymphoma immunophenotype were included: surface protein was assessed via FC and quantified with median fluorescence index ratio (MFI ratio), gene expression was evaluated by real time quantitative polymerase chain reaction (RT-qPCR) and plasmatic concentration of soluble protein (sPD-L1) measured with ELISA. Statistical analyses were performed to investigate any difference among FC immunophenotypes, updated Kiel cytological classes, and in the presence of blood infiltration.

**Results:**

Considering FC, most B-cell lymphomas (BCL) were positive, with higher MFI ratios than other subtypes (81%, median MFI ratio among positive samples = 1.50, IQR 1.21–2.03, range 1.01–3.47). Aggressive T-cell lymphomas had a lower percentage of positive samples (56%) and showed low expression (median MFI ratio in positive samples = 1.14, IQR 1.07–1.32, range 1.02–2.19), while T-zone lymphomas (TZL) were frequently positive (80%) but with low expression (median MFI ratio in positive samples = 1.19, IQR 1.03–1.46, range 1.02–6.03). Cellular transcript and sPD-L1 were detected in all samples, without differences among immunophenotypes. No correlation between results from different techniques was detected, but sPD-L1 resulted significantly increased in FC-negative lymphomas (*p* = 0.023).

**Discussion:**

PD-L1 molecule is involved in canine lymphoma pathogenesis, with differences among immunophenotypes detected by FC. Specifically, BCL have the highest expression and aggressive T-cell lymphomas the lowest, whereas TZL need further investigations.

## 1 Introduction

In both human and, more recently, veterinary oncology, therapeutic approaches for cancer are shifting from traditional chemotherapeutics to innovative strategies focused on anti-tumor immunity (1–5). The increasing interest in cancer immunotherapy has prompted a deeper exploration of molecules that influence the immune system, promoting or avoiding its reactivation and thus regulating cancer growth (6, 7).

Programmed Death-Ligand 1 (PD-L1) is a well-established immune checkpoint molecule, typically expressed by antigen-presenting cells. Its binding to Programmed Death-1 (PD-1) on T-lymphocytes initiates a signaling cascade culminating in the suppression of T-cell activation (8). When tumoral cells express PD-L1, the activation of immunosuppressive pathways through the PD-1/PD-L1 axis facilitates immune system evasion by cancer cells, thereby contributing to tumor progression (9–11). The use of inhibitors that block the interaction between PD-L1 and the PD-1 has demonstrated potential in preventing this phenomenon in several cancer types (3, 12, 13). In addition, the safety profile and clinical efficacy of an anti-canine PD-L1 monoclonal antibody were recently tested in a pilot study on 12 dogs with recurrent, metastatic, or resistant tumors following surgery, radiation, or chemotherapy, with significant results (5).

In dogs, either membrane PD-L1 (mPD-L1), mRNA and soluble protein concentration (sPD-L1) have been evaluated in various cancers, including mammary tumors, melanomas, and lymphomas (14–17). Considering canine lymphoma, Hartley and colleagues utilized flow cytometry (FC) to investigate PD-1 and PD-L1 expression both at the time of diagnosis and at relapse in nodal aspirates from dogs with B-cell lymphoma (BCL), T-cell lymphoma and healthy controls. The findings revealed an increase of PD-L1 expression in BCL, but not in T-cell lymphoma (18). In a separate study, Aresu et al. applied the RNA-scope technique to canine diffuse large B-cell lymphoma (DLBCL) histopathological sections and observed that an increasing amount of mRNA encoding for PD-L1 was associated with a worse prognosis (19). Finally, a study by Song et al. reported a significant difference in sPD-L1 plasmatic levels between healthy dogs and those with different tumors, including lymphoma (20). Notably, none of these studies assessed plasmatic, membrane protein expression, and cellular transcript in the same dog. Thus, nothing is known about the link among different forms of expression of PD-L1, leading to possible misinterpretation of results when considering studies using different techniques.

Here, our primary goal was to evaluate the different stages of PD-L1 expression in different canine lymphoma immunophenotypes. Results about transcript amount and sPD-L1 were compared to those obtained via FC in the same patient. This multiple approach aims to enhance the knowledge about the biological role of PD-L1 in dogs presenting with different lymphoma subtypes.

## 2 Materials and methods

Samples for the present study were prospectively enrolled at the FC service of the Veterinary Teaching Hospital (VTH), University of Milan, from August 2022 to December 2023. All samples were obtained for diagnostic purposes from lymph node (LN) aspirates of privately-owned dogs with suspect of lymphoma. An informed consent of the owner was always obtained. Thus, specific Ethical Committee approval to use leftover specimens for research purposes was not required (Ethical Committee decision 29 October 2012, renewed with protocol 02–2016, University of Milan).

Nodal aspirates were collected and processed for diagnostic FC as already described (21). If provided by the referring veterinarian, peripheral blood (PB) samples were processed as well, and infiltration by neoplastic cells was quantified as the percentage of cells showing the same morphological and phenotypical properties shown in the LN. A cutoff of ≥0.56% was applied to define positive PB samples. This cut-off was chosen in alignment with recommendations in the literature for DLBCL (22). However, it was uniformly applied to all samples, as no definitive analytic cutoff has been established for any other lymphoma subtype.

Cases were enrolled in the study if fulfilled the following inclusion criteria: (1) diagnosis of lymphoma based on clinical presentation, cytology and FC; and (2) adequate quality and cellularity for FC assessment of mPD-L1 expression. Cases were excluded if they were submitted for minimal residual disease (MRD) assessment in a dog already diagnosed and treated for lymphoma, or in the event of suspected relapse in a treated patient. Additionally, patients who had already undergone a chemotherapeutic agent before receiving a definitive lymphoma diagnosis were excluded.

Following FC assessment of mPD-L1 expression, when feasible, excess nodal material underwent centrifugation. The supernatant was removed, and the cell pellet was re-suspended in RNA-later (Invitrogen™ RNAlater Stabilization Solution™, catalog number AM7020) and stored at −20°C for assessment of transcript amount. If PB was available, centrifugation was performed, and plasma was separated and stored at −20°C for sPD-L1 quantification.

For each included case, the following data were recorded, if available: sex (female, spayed female, male, neutered male), breed (mixed, purebred), age (years), cytological subtype according to the updated Kiel classification (23), FC PB infiltration (presence/absence).

### 2.1 Flow cytometry

For FC assessment of mPD-L1, the LN sample was divided into three tubes, each containing 500,000 cells. Subsequently, 25 μL of a blocking solution containing 10% fetal bovine serum (FBS) and 0.2% sodium azide in RPMI 1640 (catalog number R0883) were added to each tube. The first tube served as an unstained control, the second as an isotypic control (Clone39, adivo GmbH, Germany), and the third was utilized to assess mPD-L1 expression (Clone1, adivo GmbH, Germany).

Both Clone39 and Clone1 were isolated from adivo’s proprietary fully canine antibody library using phage display methodologies (adivo GmbH, Germany). Antibodies are fully canine and belong to the IgG HC-B subtype and containing a lambda light chain. Clone1 was selected against recombinant canine PD-L1 and tested for binding recombinant antigen in Enzyme-Linked Immunosorbent Assay (ELISA) as well as for its ability to recognize native canine PD-L1 expressed on HEK-293 cells and endogenous PD-L1 on the squamous cell carcinoma cell line SCC1. In addition, the antibody was checked for unspecific binding to unrelated proteins, such as protein that were present during the antibody selection process, e.g., FBS and other blocking reagents. Moreover, binding to untransfected HEK-239 cells was tested demonstrating no stickiness/binding to unrelated proteins on the surface of cells (data not shown). Clone39 was raised against an unrelated protein by similar methodologies. Antibodies were biotinylated using No-Weigh™ EZ-Link Sulfo-NHS-LC-Biotin kit (Thermo Fisher Scientific, catalog number A39256) according to the manufacturer instructions and were tested side-by-side with the non-biotinylated variant to ensure that biotinylation did not impair antigen binding. Antibodies used in FC experiments were diluted to achieve the same concentration.

**Figure 1:**
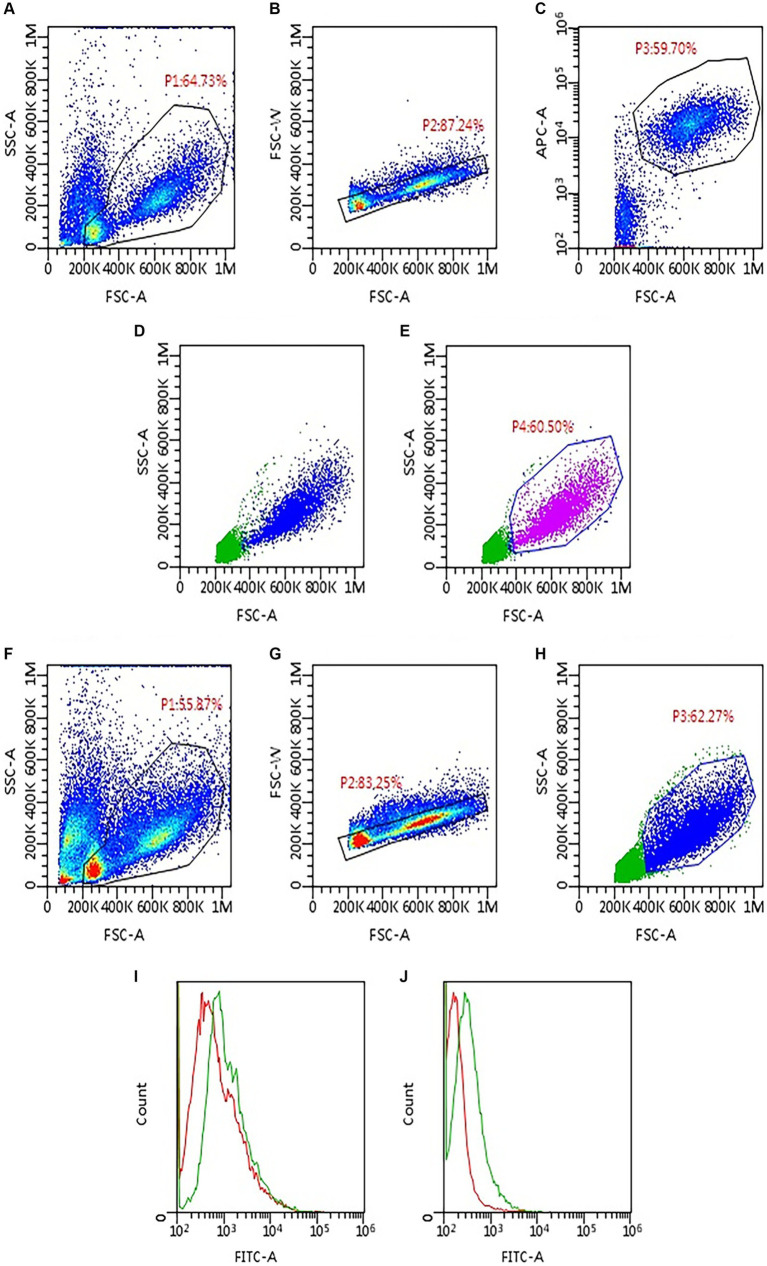
Gating strategy applied to assess PD-L1 expression via flow cytometry in 93 lymph node aspirates from dogs with lymphoma. First, samples were labeled to identify neoplastic cells based on phenotype **(A–E)**. Then, the same gating strategy was applied to the tubes incubated with anti-PD-L1 antibody and the respective isotypic control **(F–H)**. **(A)** Density plot, all events are shown; a gate (P1) was set to exclude platelet and debris. **(B)** Density plot, P1 events are shown; a gate (P2) was set to exclude doublets. **(C)** Density plot, P2 events are shown; neoplastic cells are identified based on cell size and phenotype; in the example shown, a gate (P3) was set to include only large CD21-positive cells. **(D)** Dot plot, P2 events are shown; P3 neoplastic cells are back-colored (blue dots). **(E)** Dot plot, P2 events are shown; a gate P4 was set to include only neoplastic cells based on the distribution of blue dots in panel **(D)**. **(F)** Density plot, all events are shown; P1 gate was copied and pasted from panel **(A)**, to exclude platelet and debris. **(G)** Density plot, P1 events are shown; P2 gate was copied and pasted from panel **(B)**, to exclude doublets. **(H)** Dot plot, P2 events are shown; the gate (P3) was copied and pasted from panel **(E)** (P4) to include only neoplastic cells. **(I,J)** Histogram overlay showing FITC-fluorescence in PD-L1 stained tubes (green line) compared with the respective isotypic control (red line); both samples shown were considered positive for PD-L1 expression, with lower **(I)** and higher **(J)** median fluorescence intensity (MFI) ratio.

After 10 min of incubation at room temperature and a washing step, 1 μL of an avidin kit (Avidin, Alexa Fluor™ 488 conjugate, Thermo Fisher Scientific, Waltham, Massachusetts, USA, catalog number A21370) was added to each tube. Both antibodies and avidin kit were titrated before use to determine the optimal working dilutions, utilizing a canine lymphoma cell line (CLBCL-1) previously reported to express PD-L1 (14). The tubes incubated for 10 min and finally were washed and resuspended with Phosphate Buffered Saline Solution (PBS) for acquisition at the flow cytometer. All samples were acquired with the same flow cytometer (BriCyte E6, Mindray, Shenzen, China), with constant settings and compensation matrices and data were analyzed with “MRflow” software (Mindray) by a single experienced operator (VM). Analyses were restricted to neoplastic cells, by setting a gate in an FSC versus SSC scattergram after doublets exclusion (Figure 1). The degree of mPD-L1 expression was calculated as the ratio between the Median Fluorescence Index (MFI) of the anti-PD-L1 antibody-stained tube and the isotypic control one (MFI ratio). Samples were considered positive if MFI ratio was >1, negative if it was =1.

### 2.2 Enzyme-linked immunosorbent assay (ELISA)

Plasmatic sPD-L1 levels were quantified using the specific Canine PD-L1 ELISA kit (MyBioSource Inc., San Diego, USA, catalog number MBS9349782) following the manufacturer’s protocol as suggested by Song et al. (20). This kit applies the competitive ELISA technique, using a polyclonal anti-PD-L1 antibody and a PD-L1- HorseRadish Peroxidase (HRP) conjugate, where the sample PD-L1 and PD-L1-HRP conjugate compete for binding to the anti-PD-L1 antibody site. Undiluted canine plasma samples and standard sPD-L1 samples (ranging from 10 to 0.5 ng/mL), always tested in duplicates, were incubated together with the PD-L1-HRP conjugate in an anti-sPD-L1 pre-coated plate for 1 h at 37°C. After washing the wells five times, the plate was incubated with the HRP enzyme substrate for 15–20 min at 37°C avoiding sunlight. At the end of the incubation, a stop solution (sulfuric acid, 0.18 M) was added, causing wells to change color from blue to yellow. The color intensity was measured with an ELISA microplate reader (Titertek Multiskan, Flow Laboratories, McLean, VA, USA) at 450 nm, expressing the results as Optical Density (OD). Given the competitive nature of the ELISA, the color intensity was inversely proportional to the concentration of PD-L1. The concentrations of canine plasma sPD-L1 were interpolated from the standard curve.

### 2.3 RNA extraction and RT-qPCR

Total RNA was extracted by using TRIzol reagent (Invitrogen, catalog number 15596026), according to manufacturer’s instructions for tissue samples. After quantification by QUBIT Fluorimeter, cDNA was synthesized starting from 1 μg of total RNA using the QuantiTect Reverse Transcription kit (Qiagen, catalog number 205311).

To assess the relative amounts of the PD-L1 gene expression, real-time quantitative PCR (RT-qPCR) was performed using IQ SYBR Green Supermix (BioRad, catalog number 1708882) and IQ5 Thermocycle (BioRad). GAPDH was used as housekeeping gene and RT-qPCR experiments were performed in duplicate. Quantitative RT-PCR primer sequences were as follows: primer pair PD-L1 5′-GAGAATCACAGGCACCTACAA-3′ (forward) and 5′-CGACAAGACTCCAAAGACTCAA-3′ (reverse) and primer pair GAPDH 5′-GGCACAGTCAAGGCTGAGAAC-3′ (forward) and 5′-CCAGCATCACCCCATTTGAT-3′ (reverse).

Gene expression was calculated using the formula of 2^−ΔΔCt^ (fold increase), where $\mathrm{\Delta \Delta Ct}=\Delta Ct\;\left(\mathrm{sample}\right)-\Delta Ct\;\left(\mathrm{control}\right)$ and ΔCt is the Ct of the target gene subtracted from the Ct of the housekeeping gene.

### 2.4 Statistical analysis

For statistical purposes, enrolled cases were subdivided into BCL, T-zone lymphomas (TZL) and T-cell lymphoma-Not Otherwise Specified (T-NOS) as previously reported (24). This was due to the distinctive morphology and phenotype of TZL, which predict an indolent clinical behavior (25–27). Conversely, despite marginal-zone lymphomas (MZL) being traditionally classified as an indolent subtype, they were included in the BCL group, due to their FC features, clinical behavior, and prognosis overlapping with those of DLBCL (28).

Data distribution for continuous variables was assessed with a Shapiro–Wilk test and visual inspection of histograms and q-q plots. Normally distributed data are presented as mean and standard deviation, whereas non-normally distributed data are presented as median and range. Thereafter, differences in MFI ratio, transcript amount and sPD-L1 concentration among the three lymphoma immunophenotypes (BCL, T-NOS and TZL) and among different cytological subtypes were assessed with Kruskal-Wallis or ANOVA test and appropriate post-hoc analyses (Mann Whitney test with Bonferroni correction for multiple comparisons).

Two contingency tables were prepared to calculate possible differences in FC positive and negative samples among different immunophenotypes and cytological subtypes, respectively. Fisher’s exact test was applied.

Mann–Whitney and Student t test were applied to assess differences in PD-L1 transcript amount and sPD-L1 concentration between FC positive and negative samples, and possible difference in sPD-L1 concentration between infiltrated and non-infiltrated PB samples. Spearman non-parametric correlation was applied to assess possible correlation between MFI ratio, fold increase in transcript amount, and sPD-L1 concentration. Immunophenotype was not considered for these tests.

All analyses were performed with SPSS v 28.0 for Windows, and significance was set at *p* ≤ 0.05 for all tests.

## 3 Results

In total, 93 dogs were enrolled in the present study. PD-L1 expression was investigated in all cases via FC, in 41 (44.1%) via ELISA, and in 31 (33.3%) via qPCR. Twenty-one (22.6%) cases were tested with all techniques.

Among the enrolled dogs, there were 30 (33.3%) mixed-breed and 60 (66.7%) purebred dogs, whereas the breed was not reported in 3 cases. Sex was reported for 90 dogs, including 39 (43.3%) males, 27 (30.0%) spayed females, 16 (17.8%) females, and 8 (8.9%) neutered males. The mean age at diagnosis was 8.8 ± 3.0 years.

Considering lymphoma subtype, 58 (62.4%) dogs were diagnosed with BCL, 25 (26.9%) with T-NOS, and 10 (10.7%) with TZL. In 64 cases, a cytological smear was available for review, leading to the following classification: 27 (42.2%) centroblastic polymorphic, 10 (15.6%) pleomorphic mixed, 8 (12.5%) centroblastic monomorphic, 8 (12.5%) small clear, 4 (6.2%) plasmacytoid, 3 (4.7%) pleomorphic large, 3 (4.7%) marginal zone, and 1 (1.6%) was defined as unclassified. Peripheral blood infiltration was tested in 71 cases (54 positive, 76%).

### 3.1 Flow cytometry

**Figure 2:**
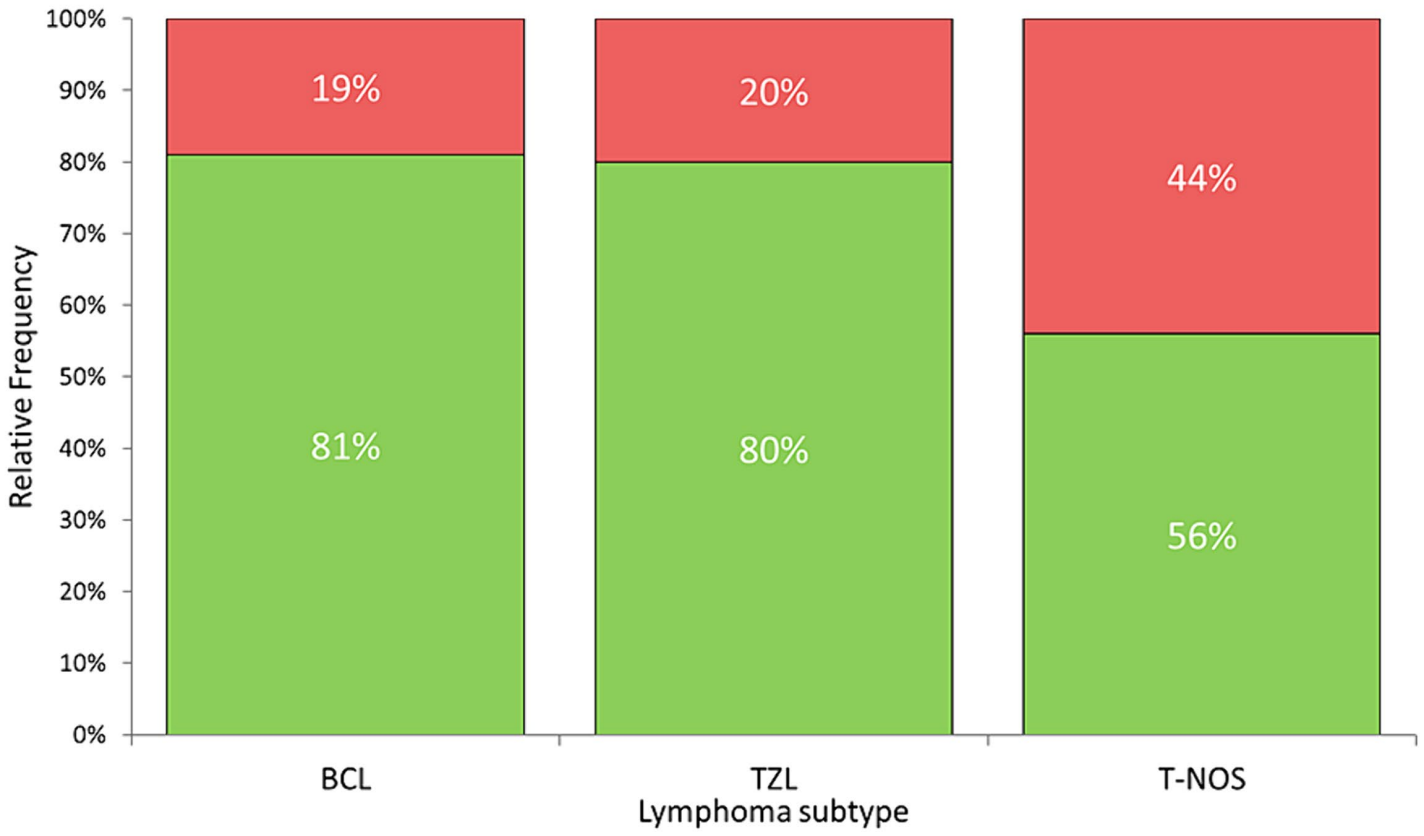
Stacked bar plots showing the percentage of samples expressing PD-L1 protein on the surface of neoplastic cells, assessed via flow cytometry on nodal aspirates from 93 dogs, according to lymphoma subtype. Green column: positive samples. Red column: negative samples. BCL, B-cell lymphoma; TZL, T-Zone lymphoma; T-NOS, T-cell lymphoma-Not Otherwise Specified.

**Figure 3:**
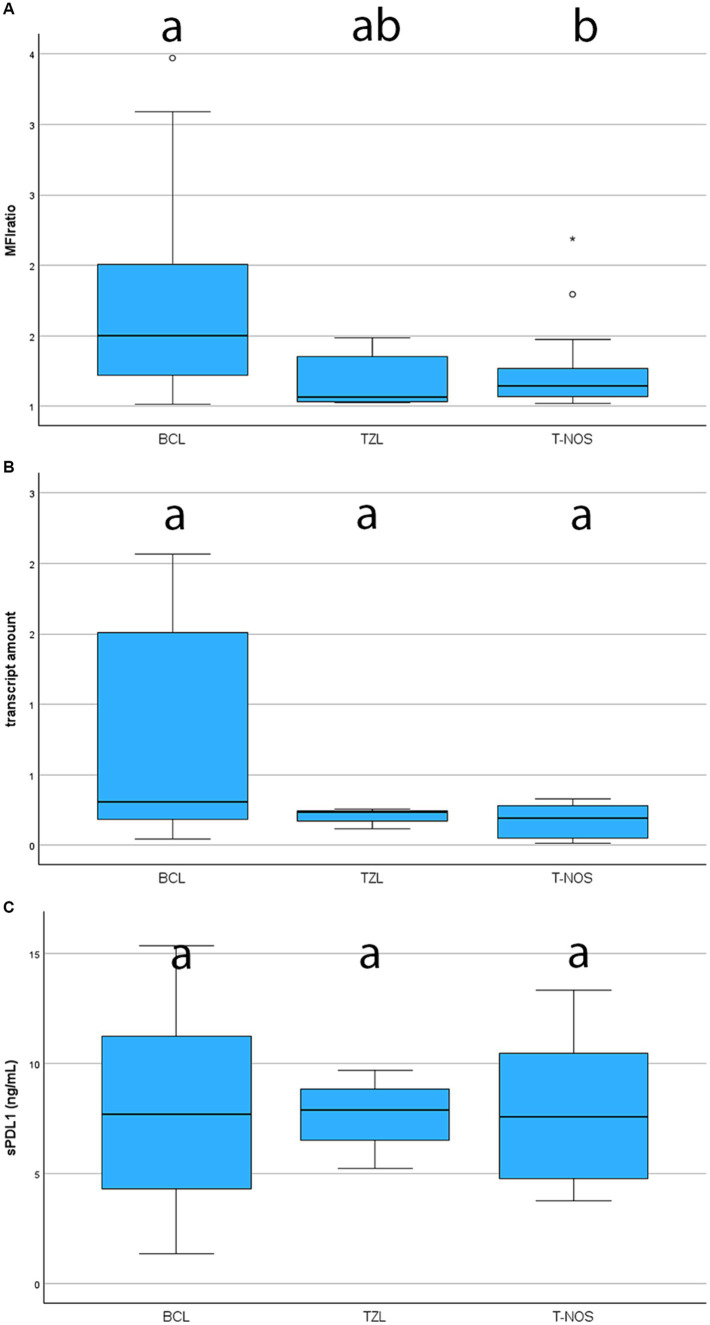
Boxplots showing the level of expression of PD-L1 assessed with different techniques, according to lymphoma subtype. BCL, B-cell lymphoma; TZL, T-Zone lymphoma; T-NOS, T-cell lymphoma-Not Otherwise Specified. The boxes with different superscript letters are significantly different. **(A)** Degree of expression on the surface of neoplastic cells, assessed via flow cytometry and quantified as the ratio between the Median Fluorescence Index (MFI) of the anti-PD-L1 antibody-stained tube and the isotypic control one (MFI ratio). Only positive samples are shown (69 dogs). One TZL case with an extremely high MFI ratio was excluded from the figure to ameliorate the graphical aspect. **(B)** Fold increase in transcript amount, assessed via RT-qPCR in 31 cases. One TZL [same case excluded in panel **(A)**] with an extremely fold increase was excluded from the figure to ameliorate the graphical aspect. **(C)** Plasmatic concentration of soluble PD-L1, assessed via ELISA in 41 cases.

Surface membrane expression of PD-L1 was detected in 69 (74.2%) samples via FC, with a median MFI ratio of 1.38 (IQR 1.12–1.80; range 1.01–6.03) among positive samples. The remaining 24 (25.8%) samples had an MFI ratio equal to 1 and were considered negative. Results among different lymphoma immunophenotypes are shown in Figures 2, 3. Based on the Fisher’s exact test, the prevalence of mPD-L1-positive samples among the three lymphoma categories was not significantly different (*p* = 0.063; Figure 2 and Table 1). Considering positive samples, the median MFI ratio significantly varied among BCL, T-NOS and TZL (*p* = 0.011; Figure 3A and Table 1). In particular, post-hoc analyses revealed a significantly higher MFI ratio in BCL than in T-NOS (*p* = 0.023), but no difference either between BCL and TZL or between T-NOS and TZL. One TZL sample had an outlier MFI ratio of 6.03. When considering Kiel subtypes, no difference in the prevalence of positive samples and median MFI ratio was detected (*p* > 0.050 for both analyses).

**Table 1:** Median MFI ratio, fold increase in the transcript amount and mean plasmatic concentration of sPD-L1 in a group of dogs with lymphoma, according to immunophenotype of neoplastic cells.

	Lymphoma immunophenotype			
	All	BCL	T-NOS	TZL
Number of FC positive samples	69 (74.2%)	47 (81.0%)	14 (56.0%)	8 (80.0%)
Median MFI ratio of positive samples	1.38 IQR 1.12–1.80 range 1.01–6.03	1.50 IQR 1.21–2.03 range 1.01–3.47	1.14 IQR 1.07–1.32 range 1.02–2.19	1.19 IQR1.03–1.46 range 1.02–6.03
Median fold increase in transcript amount	0.23 IQR 0.16–1.40 range 0.01–5.45	0.31 IQR 0.17–1.53 range 0.05–2.06	0.19 IQR 0.05–0.30 range 0.01–0.33	0.24 IQR 0.14–4.16 range 0.11–5.46
Plasmatic concentration of sPD-L1 (ng/mL) (mean ± SD)	7.88 ± 3.78	7.89 ± 4.10	7.95 ± 3.66	7.68 ± 1.83

IQR, interquartile range; SD, standard deviation; FC, flow cytometry; MFI, median fluorescence index; BCL, B-cell lymphoma; T-NOS, T-cell lymphoma-Not Otherwise Specified; TZL, T-Zone Lymphoma.

### 3.2 qPCR

PD-L1 transcript was detected in all 31 samples tested via qPCR. Among them, 19 (61.3%) were BCL, 8 (25.8%) were T-NOS and 4 (12.9%) were TZL. Overall median fold-increase in transcript amount was 0.23 (IQR 0.16–1.40; range, 0.01–5.45), with no differences among lymphoma immunophenotypes (*p* = 0.247; Figure 3B and Table 1) and Kiel subtypes (*p* = 0.597). However, none of the T-NOS samples had a fold-increase >1, differently from BCL (8 samples out of 19, 42.1%) and TZL (1 sample out of 4, 25.0%). The TZL sample with an outlier MFI ratio had a 5.45-fold increase in PD-L1 transcript amount.

### 3.3 ELISA

Plasmatic sPD-L1 was detected in all 41 samples tested via ELISA. Among them, 28 (68.3%) were BCL, 9 (22.0%) were T-NOS and 4 (9.7%) were TZL. The overall mean sPD-L1 concentration was 7.88 ± 3.78 ng/mL, with no differences observed among lymphoma immunophenotypes (*p* = 0.993; Figure 3C and Table 1) and Kiel subtypes (*p* = 0.360). Finally, sPD-L1 concentration did not vary between dogs with and without PB infiltration (*p* > 0.050).

### 3.4 Comparison among techniques

When comparing FC positive and negative samples, no difference in fold increase of transcript amount was detected (31 samples, *p* = 0.486), whereas sPD-L1 concentration was significantly higher in FC negative samples (41 samples, *p* = 0.023). Indeed, mean sPD-L1 concentration was 6.95 ± 3.39 ng/mL in FC positive samples and 10.13 ± 3.87 ng/mL in FC negative samples.

When restricting analyses to FC positive samples alone, 22 samples were included to compare MFI ratio and fold increase in transcript amount, while 29 were included to compare MFI ratio and sPD-L1. No significant correlation between MFI ratio and either fold increase in transcript amount or sPD-L1 concentration was found (*p* = 0.254 and *p* = 0.150, respectively).

## 4 Discussion

To the authors’ knowledge this is the first comprehensive assessment of PD-L1, combining evaluation of surface membrane expression, cellular transcript amount, and plasmatic concentration within the same patient in different canine lymphoma subtypes.

Overall, surface membrane protein expression by FC was detected in the majority of samples (74.2%). However, even among positive samples, the median MFI ratio was quite low, thus suggesting that canine lymphomas often express PD-L1, but with a substantial low level. This result aligns with previously published data, indicating lower PD-L1 expression in canine lymphomas compared to other cancers examined *in vitro* (29). Speculatively, this might suggest that lymphomas may exhibit a mild to low response to immunotherapy targeting PD-L1/PD-1 axis. Higher PD-L1 expression has been reported in chemotherapy-resistant than non-chemotherapy selected lymphoma cells in dogs (18). Thus, future studies including lymphoma relapses, may lead to results different from ours. In this perspective, PD-L1 immunotherapy could represent a more reliable rescue treatment than a first-line option.

Herein, BCL exhibited a significantly higher likelihood of being positive, and when positive, demonstrated a higher degree of expression compared to T-NOS, in line with the literature (14, 18). In particular, Hartley et al. (18) reported a higher expression of surface protein in neoplastic B-cells compared to their non-neoplastic counterparts, whereas this phenomenon was not observed in T-cells. Taken together, the data suggest a more significant role of PD-L1 in BCL than in T-NOS, possibly due to its suppressive activity on non-neoplastic anti-cancer T-cells (8). Concerning TZL, there is currently a lack of data on PD-L1 expression. In our case series, they showed a high prevalence of positive samples, comparable to BCL (80 and 81%, respectively) (Figure 2). Conversely, they exhibited a low MFI ratio, similar to T-NOS (Figure 3A). Most likely, the lack of statistical significance regarding TZL could be attributed to the relatively low number of cases enrolled.

Interestingly, among the 10 TZL samples assessed by FC, an outlier case with an extremely high MFI ratio and transcript amount was observed. In this dog, the concentration of sPD-L1 was only slightly over the mean value. The dog, a 13-years-old Akita Inu neutered female, received no treatment after diagnosis, and unfortunately died of causes unrelated to the neoplasm within 35 days. Consequently, no further insights could be drawn regarding the clinical relevance of the outlier PD-L1 expression. Nevertheless, reporting this case may provide valuable information related to either the biological variability of the cancer or the patient itself.

Concerning PD-L1 transcript, Ambrosius et al. (30) reported a greater expression in LN from dogs with DLBCL than from healthy controls, even if no statistical analysis was performed. Based on the lack of differences among lymphoma subtypes in the present study, it is plausible that the results obtained by Ambrosius and colleagues were associated with the presence or absence of lymphoma rather than the specific subtype considered. The same study failed to identify a prognostic role for PD-L1 transcript amount (30). Conversely, Aresu et al. found that the increased PD-L1 score quantified by RNAscope was associated with a higher risk of progression and tumor-related death in dogs with DLBCL (19). In the present study, prognostic evaluations were not performed. Thus, prognostic relevance of PD-L1 transcript amount is still controversial.

As for soluble protein, Song et al. (20) reported a difference of plasmatic concentrations between healthy and tumor-bearing dogs without assessing whether any difference existed among cancer histotypes. In the present study, no healthy dogs were included for comparison, since we only focused on lymphoma-bearing dogs. The sPD-L1 values we obtained were higher than those reported in literature (20), with no differences based on lymphoma subtype or presence of PB infiltration. This is likely due to the fact that, differently from the study of Song et al. (20), patients with comorbidities were retained in the present study, possibly leading the lymphoma-unrelated increase of sPD-L1. Inclusion of such dogs was aimed at enrolling a case-load representative of the standard population of oncological patients, with possible co-morbidities often linked to aging. Pairwise, the same cause might explain the lack of correlation between surface expression and soluble protein, as also documented in human medicine (31, 32). Unfortunately, most of the samples included in the present study were sent from referring veterinarians across Italy, and a complete anamnesis was scarcely available, preventing us from assessing the influence of other clinical variables on the concentration of sPD-L1. Nevertheless, the presence of sPD-L1, irrespective of its biological origin, should be considered in the context of immunotherapies, as it has the potential to bind and saturate the administered antibodies. Therefore, investigating its variation in a patient’s plasma and assessing it before initiating anti-PD-L1 therapies might aid in planning treatment dosage to achieve optimal effects during clinical trials.

When comparing the three techniques, the only significant result was found with sPD-L1 and FC. In particular, the sPD-L1 resulted significantly more concentrated in FC-negative cases. This could be explained by different mechanisms, including sPD-L1 release by non-neoplastic cells (i.e., dendritic cells) (33), and enzymatic cleavage of the protein on the surface of neoplastic cells (34, 35), leading to higher amount of soluble protein, while less surface protein remained.

Another interesting result is that the amount of cellular transcript does not seem to correlate with either mPD-L1 or sPD-L1. This has implications when selecting the technique to evaluate PD-L1 during future clinical trial involving immunotherapy. In the therapeutic context of human medicine, this has been already considered, and assessing surface proteins is recommended (36, 37). Relying only on mRNA expression might be misleading when trying to predict expected results of PD-L1 antibody administration.

The major pitfall of the present study is linked to the low caseload for some specific lymphoma subtypes, which might have affected the significance of statistical analyses. Anyway, the sample pool was representative of the reported prevalence of lymphoma subtypes in the canine population, with BCL being more frequent than T-NOS and TZL (38, 39). Similarly, the low number of samples analyzed by qPCR and ELISA could have impacted on the significance of those results, and it would be interesting in the future to collect a larger number of cases to eventually confirm our results.

A second limitation is that the population of neoplastic cells in FC was identified based on morphological properties (FSC) without employing a multicolor approach. This was due to the fact that the staining kit showed an undesired fluorescent signal in different channels, which was difficult to remove even with relevant compensation. The identification of neoplastic cells via FSC is usually straightforward in clinically aggressive lymphomas, mostly constituted by large cells. On the other hand, identification of neoplastic cells based on FSC is more challenging for TZL and it is possible that a proportion of residual lymphocytes has been included in the analysis, while a proportion of neoplastic cells has been excluded. However, such a condition is not likely to have grossly biased MFI ratio analysis, since TZL cells represented the vast majority of the population within each sample, and their FSC is actually higher than the one of residual lymphocytes (40), although perhaps less immediately identifiable by unexperienced operators. No further small cell lymphoma was included in the present study.

Lastly, clinical and follow-up data, when retrieved, were fragmentary, thus preventing us from including them. Future studies assessing correlations between the variables evaluated and prognosis are warranted.

## 5 Conclusion

PD-L1 is often expressed on cells’ surface in canine lymphomas, typically with low intensity. BCL are frequently positive and have the highest amount of surface protein compared to TZL and T-NOS. Although cellular transcript resulted present in all samples, no correlation with lymphoma categories or other techniques was found. sPD-L1 is higher in the samples with no surface protein expression, as possible result of the cleavage and release of surface proteins in the plasma.

Future studies should carefully consider the technique for assessing PD-L1 expression, since results are not correlated and interchangeable. This consideration is crucial when admitting dogs with lymphoma to immunotherapies targeting the surface membrane PD-L1.

## Data availability statement

The raw data supporting the conclusions of this article will be made available by the authors, without undue reservation.

## Ethics statement

The requirement of ethical approval was waived by Ethical Committee, University of Milan, Italy for the studies involving animals because all samples were obtained for diagnostic purposes from lymph node (LN) aspirates of privately-owned dogs with suspect of lymphoma. An informed consent of the owner was always requested. Thus, specific EC approval to use leftover specimens for research purposes was not required (EC decision 29 October 2012, renewed with protocol 02-2016, University of Milan). The studies were conducted in accordance with the local legislation and institutional requirements. Written informed consent was obtained from the owners for the participation of their animals in this study.

## Author contributions

AU: Data curation, Investigation, Validation, Writing – original draft. LC: Investigation, Writing – review & editing. PD: Investigation, Writing – review & editing. RM: Investigation, Writing – review & editing. LA: Writing – review & editing. PM: Visualization, Writing – review & editing. FS: Writing – review & editing. FR: Writing – review & editing. DS: Writing – review & editing. SC: Writing – review & editing. VM: Formal analysis, Investigation, Project administration, Supervision, Writing – review & editing.
